# Therapeutic Prospects of Polysaccharides for Ovarian Cancer

**DOI:** 10.3389/fnut.2022.879111

**Published:** 2022-04-07

**Authors:** Kaili Wang, Mengcheng Cai, Shuai Sun, Wen Cheng, Dongxia Zhai, Zhexin Ni, Chaoqin Yu

**Affiliations:** Department of Traditional Chinese Gynecology, The First Affiliated Hospital of Naval Medical University, Shanghai, China

**Keywords:** polysaccharides, anticancer, ovarian cancer, combination treatment, platinum, drug resistance

## Abstract

Ovarian cancer (OC) is ranked as the leading cause of death among cancers of the female reproductive tract. First-line platinum treatment faces the severe challenges associated with the patient relapse and poor prognosis. Thus, it is imperative to develop natural antitumor drugs for OC with high efficacy. Natural polysaccharides have significant biological activities and antitumor effects. Our work has demonstrated that polysaccharides play key roles by inhibiting the cell proliferation and growth, regulating the tumor cell cycle, inducing apoptosis, suppressing the tumor cell migration and invasion, improving the immunomodulatory activities, and enhancing the efficacy of chemotherapy (cisplatin) in OC, which provide powerful evidence for the application of polysaccharides as novel anticancer agents, supplementary remedies, and adjunct therapeutic agents alone or in combination with cisplatin for preventing and treating the OC.

## Introduction

Ovarian cancer (OC) is the leading cause of death due to female gynecological cancers, is characterized by high lethality and a high death incidence ratio (1, 2), and its early detection is not readily achieved (3, 4). Among all malignant gynecological lesions, the fatality rate ranks first, posing a great threat to the health of patients. Currently, chemotherapy is one of the major treatments for OC, and platinum-related chemotherapy is the first-line treatment for OC (1, 2). However, most chemotherapeutic agents inhibit cancer cells but also exhibit undesirable side effects on normal tissues, resulting in a decline in patient immune function and causing serious effects on the human body, and the treatment is expensive (3, 4). To improve the efficacy of chemotherapy, it is imperative to identify and develop natural antitumour drugs with high efficacy and low toxicity (1, 5–7). In addition, cisplatin resistance and sensitivity are gradually becoming severe problems in OC treatment; these issues mainly account for the patient relapse and poor prognosis, and ~65–80% of patients experience recurrence within the first 5 years (1, 6). Therefore, it is critical to develop novel agents and therapeutic strategies to enhance the clinical efficacy and inhibit drug resistance when treating OC.

Polysaccharides are natural polymers formed by covalently linking multiple monosaccharides through glycosidic bonds and have minor side-effects, which are one of the four basic substances that constitute life and are widely distributed in microorganisms, plants, and animals (8, 9). Their structures and molecular size always in range from linear to highly branched (10, 11), which include storage polysaccharides and structural polysaccharides (8); and natural polysaccharides display both branched and linear polymer architectures, such as starch, glycogen, cellulose, and chitin (8, 10, 11). In clinic, polysaccharides from medicinal plants mainly include ginseng polysaccharide, astragalus polysaccharide, Ganoderma lucidum polysaccharide, lotus polysaccharide, lentinus edodes polysaccharide, and Ruminococcus mushroom polysaccharide, which have attracted a lot of attention due to their significant anti-tumour bioactivities (8, 12).

An increasing number of studies have suggested that polysaccharides have a wide range of biological activities and pharmacological effects *via* immunoregulation (13–15), anticancer activity (16, 17), and antioxidation (14, 17). Polysaccharides not only regulate the metabolism and immune function, but also have antitumour, antiviral, antioxidation, antiaging, and hypoglycaemic effects (8, 9, 12). Because of the significant antitumour activity, high efficacy, low toxicity, wide availability, and low price, polysaccharides have received increasing attention from researchers worldwide (18, 19). Although polysaccharides have been widely studied by researchers, it has not been clearly determined whether polysaccharides can be used to treat OC as candidate drugs or adjuvant agents combined with cisplatin chemotherapy, and no summarized reports or analyses have been conducted to assess the therapeutic prospects of polysaccharides as a treatment.

Therefore, based on the literature, we summarized and analyzed the pharmacological effects and mechanisms of polysaccharides in OC to provide a theoretical basis for subsequent preparation, development, clinical research, and applications. Moreover, we systematically explored and discussed the therapeutic effects and clinical prospects of polysaccharides in pretreating and treating OC, which has further provided powerful support and evidence.

## Effects of Polysaccharides on OC

### Effects of Polysaccharides on Cell Proliferation and Apoptosis

**Figure 1:**
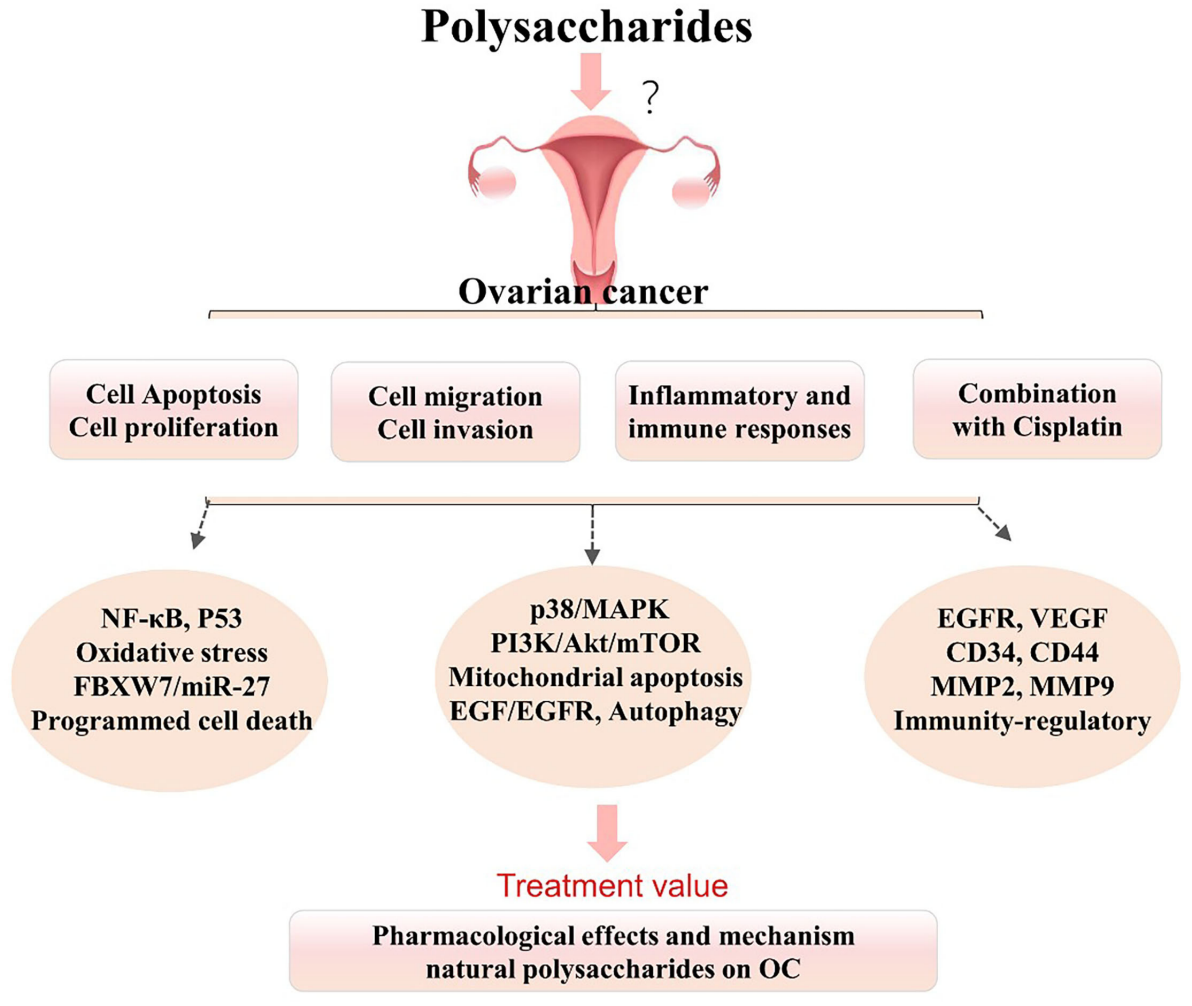
Summarized effects and mechanisms underlying natural polysaccharides in the treatment of ovarian cancer (OC). Polysaccharides play vital roles in the treatment of OC *via* multiple pathways and molecules, but it remains uncertain whether polysaccharides can be used in the clinical treatment of OC. It is uncertain or not completely verified. BAX, BCL2-associated X protein; EGFR, epidermal growth factor receptor; FBXW7, F-box, and WD-40 domain protein 7; MMP-2/9, matrix metalloproteinase 2/9; MAPK, mitogen-activated protein kinase; NF-κB, nuclear factor kappa B; OC, ovarian cancer; PI3K, phosphatidylinositol 3-kinase; P53, tumor suppressor gene-53; VEGF, vascular endothelial growth factor.

Apoptosis is a form of programmed death that is highly regulated and plays an important role in cancer treatment, and it is a popular target of many treatment strategies (7, 20). The occurrence, development, and regression of OC are closely related to apoptosis, and inducing apoptosis in cancer cells is a research hot spot and a vital strategy for tumor treatment (2, 21). The previous studies (Figure 1) have shown that the natural polysaccharides exert anticancer effects on OC models by regulating the tumor cell cycle, inhibiting cell proliferation, and inducing the apoptosis *in vitro* and *in vivo* (22–28).

The BPP is a natural polysaccharide found in *Balanophora polyandra* that can inhibit the proliferation and growth of ovarian tumors formed by SKOV3 (human ovarian carcinoma cells) cells *in vitro* and in rats with OC. These results suggest that BPP regulates the S phase of cell cycle arrest by decreasing the expression levels of cyclin A and cyclin-dependent kinase 2 (CDK2). The BPP triggers the apoptosis in OC cells in a concentration-dependent manner, and this induction of apoptosis involves the downregulation of B-cell lymphoma-2 (BCL-2) in A2780 and OVSAHO cells (22). In addition, the noted antitumour effects of BPP are related to the tumor suppressor gene-53 (also known as P53) pathway (22).

The APS is a natural antioxidant polysaccharide obtained from *Astragalus membranaceus* that exerts antiproliferative and proapoptotic effects on OC. APS inhibits not only proliferation, but also invasion and migration in OC cells. APS treatment induces apoptosis in OC cells by downregulating the expression of miR-27a, which was confirmed by RNA sequencing and overexpression/knockdown cells. F-box and WD-40 domain repeat-containing protein 7 (FBXW7) is a direct target of miR-27a and can reverse the antitumor effect of APS, indicating that APS induces apoptosis in OVCAR-3 cells *via* the FBXW7/miR-27 pathway (24).

*Polygala tenuifolia* polysaccharide (PTP) is a natural polysaccharide isolated from the roots of *Polygala tenuifolia* that plays a key role in inhibiting OC. On the one hand, PTP significantly suppresses the growth of SKOV3 cells *in vitro* and in OC model rats, inhibits the proliferation of SKOV3 cells in a concentration-dependent manner (23, 29), decreases tumor growth in nude mice, rapidly exhausts glutathione (GSH) level (23), and promotes the production of intracellular reactive oxygen species (ROS) (23, 30). On the other hand, PTP inhibits the proliferation of OVCAR-3 cells, induces cell death by arresting the cell cycle at the G0/G1 phase, and suppresses the growth in OC cells (27, 28). These effects on human OC may be associated with the increased expression of mitochondrial proteins (BCL2-associated X [BAX] protein, caspase-3/9, and cytochrome c), nuclear factor kappa B (NF-κB), and the programmed cell death pathway (27, 28).

Furthermore, MDP-A1 and MDP-A2 are acidic polysaccharides, isolated from the rhizome of *Menispermum dauricum* (MW 9.1 × 10^4^ – 5.8 × 10^4^ Da), significantly inhibit the proliferation of human SKOV3 cells in a concentration-dependent manner, increase the number of apoptotic cells, and reduce the expression of caspase-3 and caspase-8; these effects were further observed in mice (25). In addition, PPC has negligible cytotoxicity on fibroblasts, is a type of polysaccharide–protein complex, and can induce the death in tumor cells through apoptosis and necrosis (26), which may be addressed with chemical modifications.

Overall, these findings suggest that various polysaccharides from natural plants can inhibit cell growth and proliferation and induce apoptosis in OC cells *via* the mitochondrial apoptosis pathway, the FBXW7/miR-27 pathway, the NF-κB and programmed cell death pathways, and oxidative stress regulation and the P53 pathway and they may be beneficial as potential therapeutic agents for the treatment of OC.

### Effects of Polysaccharides on Cell Migration and Invasion

Cancer cell migration and invasion in tissue and the vasculature are the initial and dynamic steps that play an important role in the tumor progression and cancer metastasis (31, 32). Thus, it is crucial to develop novel treatment strategies to inhibit tumor cell migration and invasion, and get prevention from this life-threatening phenomenon (31, 32). In addition to suppressing tumor cell growth and proliferation (22, 24), natural polysaccharides inhibit OC tumor cell migration and invasion (33–35).

The *Bangia fuscopurpurea* (BFP) polysaccharide is a novel polysaccharide extracted from *Bangia fuscopurpurea* that has been confirmed to increase the accumulation of ROS, reduce mitochondrial membrane potential, and thus induce apoptosis in A2780 OC cells. Moreover, BFP polysaccharide inhibits the migration and invasion of OC cells. Further results show that BFP polysaccharide induces cell death by activating the autophagy (Beclin1-LC3-P62) pathway and promotes the mitochondrial apoptosis pathway by downregulating BCL2 expression, upregulating BAX protein expression, and increasing cytochrome C release in A2780 cells (34). These findings demonstrate that BFP polysaccharide may be useful for developing functional foods for adjuvant anticancer treatment.

The SIP-SII is a sulfated derivative polysaccharide from *Sepiella maindroni* that significantly inhibits the tumor growth and metastasis (35). First, the migration and invasion of SKOV-3 cells are notably inhibited by SIP-SII. Second, SIP-SII is transported and located on the cell membrane, downregulates the expression of epidermal growth factor receptor (EGFR) and matrix metalloproteinase 2 (MMP-2), attenuates EGF-induced EGFR phosphorylation, and suppressed OC cell migration. Additionally, SIP-SII significantly inhibited the p38/mitogen-activated protein kinase (MAPK) and phosphatidylinositol 3-kinase (PI3K)/Akt/mTOR signaling pathways in SKOV-3 cells. These findings demonstrate that SIP-SII has potential inhibitory effects on tumor metastasis in OC, which are associated with regulating the p38/MAPK and PI3K/Akt/mTOR signaling pathways (35).

The CPPA is an acidic polysaccharide isolated from *Codonopsis pilosula* that inhibits the proliferation of HO-8910 cells (human epithelial OC cells) *in vitro*. CPPA treatment significantly inhibits invasion, migration, and adhesion in tumor cells by down regulating the CD44 expression (33), indicating that CPPA may be a potential candidate for the prevention of tumor metastasis. In addition, APS and BPP inhibit migration and invasion in OC cells *via* the FBXW7/miR-27a and P53-mediated pathways (22, 24).

In summary, these results (Figure 1) further suggest that polysaccharides not only play vital roles in the induction of OC cells, but also show marked regulatory effects on tumor metastasis (cell migration and invasion) in OC. These polysaccharides have great potential as novel agents against tumor metastasis *via* the p38/MAPK and PI3K/Akt/mTOR signaling pathways, the mitochondrial apoptosis pathway, the EGF-EGFR and MMP2 pathways, and the FBXW7/miR-27a and P53 pathways.

### Effects of Polysaccharides on Inflammatory and Immune Responses

Inflammatory and immune responses play vital roles at different stages of tumor development, such as initiation, invasion, proliferation, malignant conversion, and metastasis (36, 37). Therefore, it is of great importance to develop new agents with immunomodulatory activities to improve the efficacy of chemotherapy (cisplatin). Recent studies indicate that various polysaccharides have antioxidant and immunoregulatory effects on OC (29, 33, 38–40).

The PSP is a polysaccharide extracted from fresh purslane that significantly inhibits superoxide anion, 2,2-Diphenyl-1-picrylhydrazyl (DPPH) free radical, hydroxyl radicals, and nitric oxide (NO) in a dose-dependent manner. The PSP effectively increased splenocyte, thymocyte, and T and B lymphocyte proliferation and also improved the function of red blood cells. These findings support the use of polysaccharides to treat OC, and these effects are mediated by scavenging accumulating free radicals and enhancing immunity (29).

*Polygala tenuifolia* polysaccharide is known to inhibit the growth and proliferation of OC cells (23, 29, 30), which are at least partially related to inflammation and the immune response. First, PTP increases serum vascular endothelial growth factor (VEGF) and EGFR levels and downregulates the transcript and protein levels of EGFR, VEGF, and CD34 *in vivo* (29). Moreover, PTP regulates the NF-κB pathway in SKOV3 cells (27, 28). *In vivo* and *in vitro* evidence suggests that PTP is a powerful adjuvant therapy for patients with OC at an advanced stage.

BPS is a polysaccharide isolated from basil that can inhibit the invasion of SKOV3 cells. In contrast, coadministration of BSP and curcumin significantly suppressed the invasion of SKOV3 cells and immature and mature human monocyte-derived dendritic cells (DCs) and reduced the mRNA and protein expression of CD44 and MMP-9 in SKOV3 cells, indicating that curcumin and BPS regulated the invasion of SKOV3 cells and DCs by distinctly downregulating osteopontin (OPN), CD44, and MMP-9 expression. Moreover, CAPP also inhibited the invasion, migration, and adhesion of tumor cells by regulating CD44 (33, 40). *Ganoderma lucidum* and polysaccharides enhance immune functions and inhibit cancer cell growth (41).

Therefore, curcumin and these polysaccharides (CAPP, PSP, and BPS) may be suitable candidates for OC immunotherapy (Figure 1). These results suggest that these polysaccharides can be used as adjunct supplementary agents in chemotherapy and immunotherapy to threaten OC *via* immunoregulation. However, the underlying mechanisms and pathways of polysaccharide-related immunotherapy remain unclear.

## Effects of Combination Treatment With Polysaccharides and Cisplatin on OC

**Figure 2:**
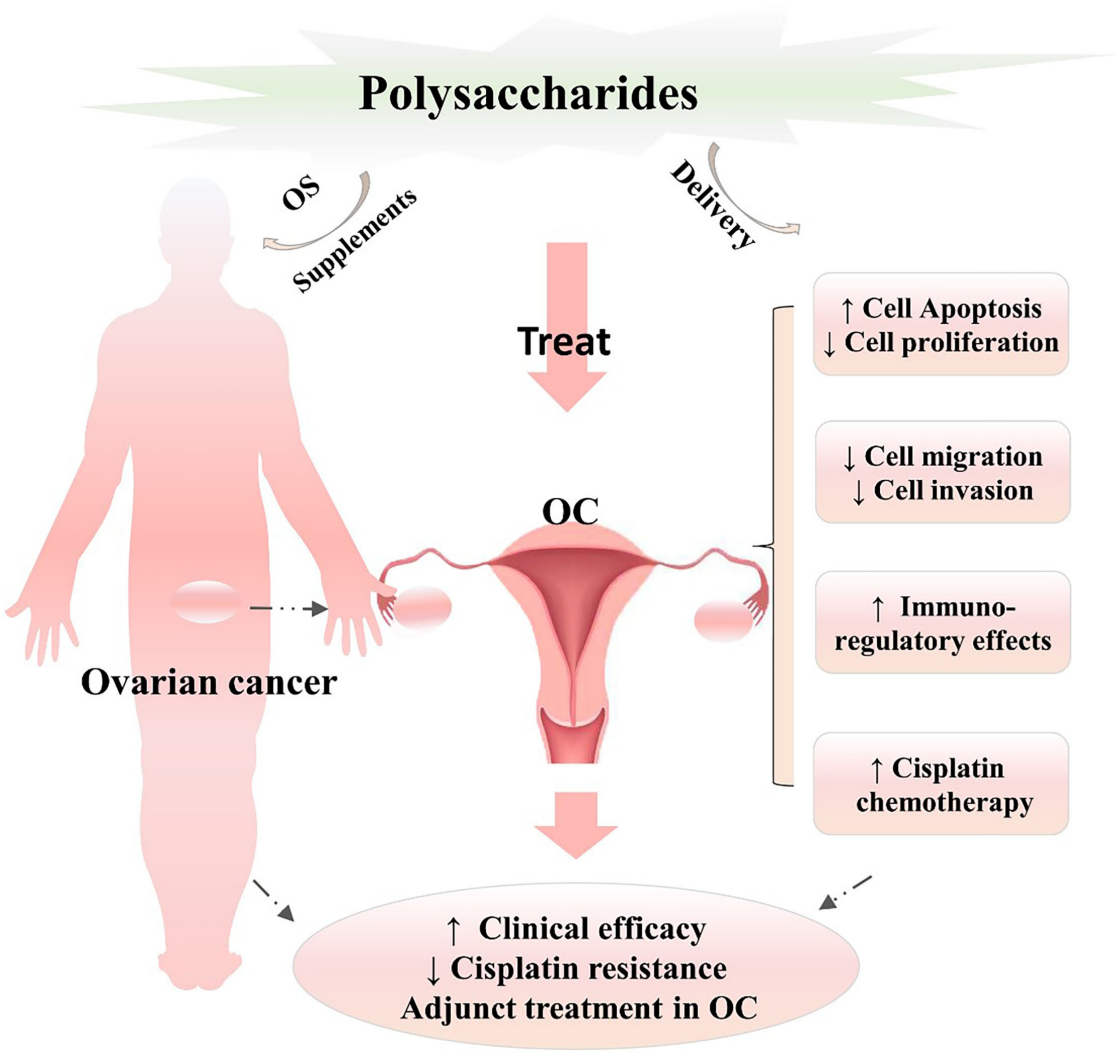
Analysis of the therapeutic prospects of natural polysaccharides in the treatment of OC. Polysaccharides play vital roles in inhibiting cell proliferation and growth, regulating the tumor cell cycle, inducing cell apoptosis, suppressing tumor cell migration and invasion, improving immunomodulatory activities, and enhancing the efficacy of chemotherapy in OC and can be developed as adjunct agents or supplementary treatments in combination with cisplatin, which needs to further develop in the process of polysaccharide healthcare products in future work. OC, ovarian cancer; ↑, significantly enhance or improve; ↓ significantly downregulate or inhibit.

Ovarian cancer is characterized by high lethality and an impressive death-to-incidence ratio. Currently, platinum-based therapies are the first-line treatment of OC (1, 6), but platinum sensitivity and drug resistance are huge challenges in the clinical treatment of OC, causing the majority of patients to relapse and have a poor prognosis. Thus, it is of great importance to develop new adjunct agents and supplemental therapies for OC by enhancing therapeutic effects and inhibiting drug resistance in combination with platinum. Recent studies (Figures 1, 2) have shown that polysaccharides can increase sensitivity and ameliorate resistance to cisplatin treatment of OC and thus have great potential to enhance chemosensitivity (39–43).

The APS is a natural polysaccharide that not only exerts antiproliferative and proapoptotic effects on various cancers (24), but also enhances the sensitivity of cancer cells to chemotherapeutic drugs (42). Treatment with APS and cisplatin synergistically enhanced the inhibitory effects of cisplatin on cell viability and promoted cisplatin-induced apoptosis. The enhanced effects mainly stemmed from the regulation of BCL2, BAX, and caspase 3 expressions *via* the c-Jun amino terminal kinase (JNK1/2) signaling pathway, which was further confirmed by related inhibitors (42).

The DDAP is a novel polymeric platinum compound that conjugates the platinum analog to aspartate chondroitin, which is regarded as a combined complex of polysaccharides and platinum. On the one hand, DDAP improves platinum solubility, enhances drug delivery, and increases cellular uptake of DDAP in platinum-resistant OC cells, which reduces systemic toxicity and produces more efficacious outcomes. On the other hand, DDAP significantly inhibits cell growth, especially at lower doses, induces apoptosis, and arrests cells in the S-phase (43). The related mechanisms may be associated with the caspase 3 pathway, the upregulation of p21, and the maintenance of cyclin A (43).

The PS_K is protein-bound polysaccharides collected by Coriolus versicolour QUEL that has similar activities as superoxide dismutase (SOD) (39); the coadministration of PS_K and cisplatin can prevent cisplatin-associated cytotoxicity in NRK-49F cells. Cisplatin increases lipid peroxides, decreases SOD activity at the IC_50_, and markedly inhibits proliferation in each cell line. When administered with PS_K, these effects were augmented in H4-II-E and OC cells but diminished in the NRK-49F cell line, indicating that cisplatin combined with PS-K has more significant anticancer effects on OC patients than monotherapy (39).

In addition, *Ganoderma lucidum* enhances immune functions and exerts its antitumour effects on various cancers (41). Polysaccharides and triterpenes are biologically active constituents of *Ganoderma lucidum*; thus, polysaccharides of *Ganoderma lucidum* have great potential in suppressing cell growth, inducing apoptosis in OVCAR-3 cells, inhibiting the disruption of cell cycle progression by downregulating cyclin D1, and regulating antioxidative/detoxification activity *via* the nuclear factor E2-related factor 2 (Nrf2)-mediated signaling pathway, such as the antioxidant NAD(P)H, SOD, catalase, quinone oxidoreductase 1 (NQO1), and glutathione S-transferase P1 (41). These chemopreventive effects provide powerful support for the use of *Ganoderma lucidum*, and these polysaccharides can be developed as adjunct supplementary agents in combination with cisplatin chemotherapy and have great clinical prospects in OC treatment.

## Conclusions and Prospects

Ovarian cancer is ranked first among female gynecological cancers (1). Because patients relapse and develop resistance to first-line cisplatin chemotherapy (6, 44, 45), enhancing the clinical efficacy (sensitivity), and ameliorating cisplatin drug resistance are increasingly major problems. Fortunately, natural active polysaccharides provide an alternative OC treatment as adjunct therapeutic agents or in combination with cisplatin. In the present work (Figure 2), we systematically explored and analyzed the therapeutic effects and clinical prospects of polysaccharide pretreatment and treatment as OC therapies, further providing powerful support and evidence for this viewpoint. In addition, hyaluronan, a high-MW polysaccharide, can be used for magnetic resonance imaging (MRI) visualization of hyaluronidase in OC (46); glycosaminoglycan motifs may form a new class of biomarkers for OC, as indicated here for the GD3G7 epitope in OC tissue (47).

Taken together, the currently available data and results (Figure 2) suggest that natural polysaccharides have significant biological activities and pharmacological effects and play vital roles in inhibiting the cell proliferation and growth, regulating the tumor cell cycle, inducing cell apoptosis, suppressing tumor cell migration and invasion, improving immunomodulatory activities, and enhancing the efficacy of chemotherapy (cisplatin) in OC. However, because of the lack of clinical testing and clinical data to further support the clinical therapeutic efficacy and safety of natural polysaccharides in OC, further clinical trials and evidence are essential. At present, functional drug delivery systems combining polysaccharides and cisplatin are rare in the clinic, and active polysaccharide-enriched supplements are limited in the market. Some polysaccharide complexes with biological functions, which have health and medical value and broad prospects, need to be further developed.

## Author Contributions

KW and MC designed the review. KW, MC, and SS wrote the manuscript. WC, DZ, and KW helped in creating the figures and revising the manuscript. ZN and CY were mainly responsible for supervising the work and managing related projects. All authors discussed, edited, approved the final version, and agreed to be accountable for the content of this work.

## Funding

This work was funded by the National Natural Science Foundation of China (82074206 and 81973896), the Shanghai 3-Year Action Plan for Traditional Chinese Medicine [ZY(2018-2020)-FWTX-1107], the Leading Project of Traditional Chinese Medicine of Shanghai Science and Technology Committee (19401930200), and the China Postdoctoral Science Foundation (2020M681337).

## Conflict of Interest

The authors declare that the research was conducted in the absence of any commercial or financial relationships that could be construed as a potential conflict of interest.

## Publisher's Note

All claims expressed in this article are solely those of the authors and do not necessarily represent those of their affiliated organizations, or those of the publisher, the editors and the reviewers. Any product that may be evaluated in this article, or claim that may be made by its manufacturer, is not guaranteed or endorsed by the publisher.
